# Generalizable and robust deep learning algorithm for atrial fibrillation diagnosis across geography, ages and sexes

**DOI:** 10.1038/s41746-023-00791-1

**Published:** 2023-03-17

**Authors:** Shany Biton, Mohsin Aldhafeeri, Erez Marcusohn, Kenta Tsutsui, Tom Szwagier, Adi Elias, Julien Oster, Jean Marc Sellal, Mahmoud Suleiman, Joachim A. Behar

**Affiliations:** 1 Faculty of Biomedical Engineering, Technion-IIT, Israel; 2 Department of Cardiology, Centre hospitalier Universitaire de Nancy, Nancy, France; 3 Department of Cardiology, Rambam Medical Center and Technion The Ruth and Bruce Rappaport Faculty of Medicine, Haifa, Israel; 4 Department of Cardiovascular Medicine, Faculty of Medicine, Saitama Medical University International Medical Center, Saitama, Japan; 5 Mines Paris, PSL Research University, Paris, France; 6 IADI, U1254, Inserm, Université de Lorraine, Nancy, France; 7 CIC-IT 1433, Université de Lorraine, Inserm, CHRU de Nancy, Nancy, France; 8 Present Address: IADI, U1254, Inserm, Université de Lorraine, Nancy, France

**Keywords:** Atrial fibrillation, Diagnostic markers

## Abstract

To drive health innovation that meets the needs of all and democratize healthcare, there is a need to assess the generalization performance of deep learning (DL) algorithms across various distribution shifts to ensure that these algorithms are robust. This retrospective study is, to the best of our knowledge, an original attempt to develop and assess the generalization performance of a DL model for AF events detection from long term beat-to-beat intervals across geography, ages and sexes. The new recurrent DL model, denoted ArNet2, is developed on a large retrospective dataset of 2,147 patients totaling 51,386 h obtained from continuous electrocardiogram (ECG). The model’s generalization is evaluated on manually annotated test sets from four centers (USA, Israel, Japan and China) totaling 402 patients. The model is further validated on a retrospective dataset of 1,825 consecutives Holter recordings from Israel. The model outperforms benchmark state-of-the-art models and generalized well across geography, ages and sexes. For the task of event detection ArNet2 performance was higher for female than male, higher for young adults (less than 61 years old) than other age groups and across geography. Finally, ArNet2 shows better performance for the test sets from the USA and China. The main finding explaining these variations is an impairment in performance in groups with a higher prevalence of atrial flutter (AFL). Our findings on the relative performance of ArNet2 across groups may have clinical implications on the choice of the preferred AF examination method to use relative to the group of interest.

## Introduction

Atrial fibrillation (AF) is the most prevalent heart arrhythmia^1,2^. It is associated with a fivefold increase in stroke incidence and a 3.5-fold mortality risk increase^3^. Across geography, ages and sexes AF has been reported to have varying prevalence^1,2,4–6^. Machine learning presents a unique opportunity to provide an accurate automated diagnosis of AF. Yet, these models must demonstrate generalizability to external datasets integrating a range of population samples. We searched PubMed up to May 24th, 2022, for research articles containing the terms “(deep learning or convolutional neural network)” AND “(atrial fibrillation or atrial flutter)” AND “(generalization across geography or generalization across age or generalization across sex)”, without any date or language restrictions. We also reviewed reference lists of eligible articles. Our search did not identify any previous studies assessing deep learning (DL) algorithms generalization across geography, ages and sexes for the task of AF detection. We hypothesize that a DL approach could outperform the classical feature engineering approaches by leverage a large training set and learning a valuable representation of the beat-to-beat interval dynamic. This work addresses the challenge of generalization performance of a new DL AF event detection algorithm.

## Results

### Datasets

**Fig. 1:**
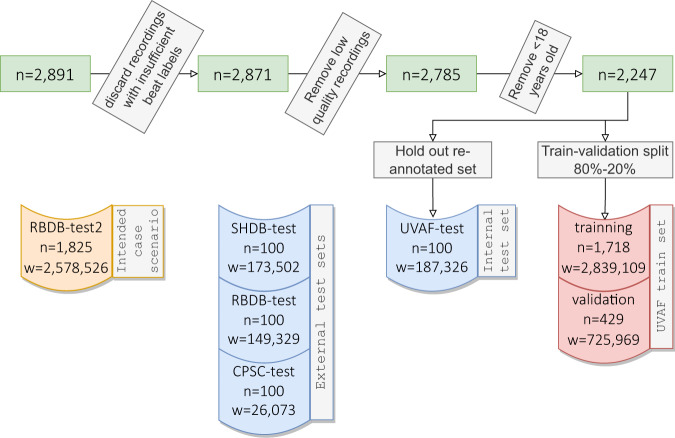
Data exclusion and stratification. Description of the exclusion process applied to the UVAF database and of all datasets used in this research.

The University of Virginia dataset (UVAF) from the USA^7,8^ was used to train the DL models. UVAF consists of a dataset of 2147 patients totaling 51,386 h of annotated continuous electrocardiogram (ECG). The model’s generalization was then evaluated on manually annotated events in test sets from four centers (USA, Israel, Japan and China) totaling 402 patients. These test datasets are denoted UVAF-test, RBDB-test, SHDB-test, CPSC-test, (Table 1). Finally, the new DL model was validated on a retrospective dataset of 1825 consecutives Holter recordings from the Rambam Hospital Holter clinic, Haifa, Israel (denoted RBDB-test2). Overall, the datasets totaled 4,298 ECG Holter recordings and over 99,705 h of continuous data. Annotations for AF and atrial flutter (AFL) were grouped under a single label denoted AF_l_ for the experiments and similar to previous works^7–10^. Holter recordings of patients under the age of 18 years were excluded since this research focused on AF diagnosis in adults. Furthermore, corrupted recordings were also excluded. Overall, out of 2891 UVAF-train recordings, 106 (3.7%) were excluded due to low quality or missing annotations as summarized in Fig. 1. No recordings were excluded from the test sets. The PhysioZoo software^11,12^ was installed on a server within the Technion domain and accessed remotely by a fellow in cardiology annotator (MA), who annotated the test set Holter ECGs. Supraventricular arrhythmias were divided and annotated, using our annotation protocol, into five categories: (1) AF, (2) AFL, (3) atrial tachycardia (AT), (4) Other supraventricular tachycardias such as Wolf-Parkinson-White and intranodal tachycardias and (5) other, such as NSR, that were not labeled. Reading time by the fellow (MA) was estimated to be on average 45 min per 24 h Holter recording.

**Table 1:** Description of the population samples.

	Origin	Recordings, n	Patients, p	Age (yrs) μ (Q1-Q3)	Female, *n* (%)	Recordings length μ (Q1-Q3)(hrs)	AF prevalence, *n* (%)
Geography							
UVAF-train	USA	2147	1970	57.0 (39.0–71.0)	1169 (54.4)	24.0, (24.0–24.0)	151 (7.0)
UVAF-test	USA	100	100	69.0 (58.8–76.2)	50 (50.0)	24.0 (24.0–24.0)	73 (73.0)
SHDB-test	Japan	100	100	69.5 (62.0–75.2)	45 (45.0)	24.00 (23.9–24.0)	79 (79.0)
RBDB-test	Israel	100	100	69.5 (56.8–78.0)	50 (50.0)	24.8 (21.7–25.2)	57 (57.0)
CPSC-test	China	102	102	69.0 (59.0–76.0)	41 (40.0)	0.2 (0.1–0.5)	51 (50.0)
Combined test set	USA, Japan, Israel, China	402	402	69.0 (59.0–77.0)	186 (46.3)	24.0 (1.5–24.0)	260 (64.7)
Sex							
Female	USA, Japan, Israel, China	186	186	68.0 (58.0–77.0)	186 (100.0)	12.9 (0.34–12.9)	111 (59.7)
Male	USA, Japan, Israel, China	215	215	70.0 (59.0–76.0)	215 (100.0)	11.2 (0.2–11.2)	148 (68.8)
Ages							
≤60	USA, Japan, Israel, China	116	116	53.0 (40.0–56.0)	59 (50.8)	20.7 (0.3–20.7)	47 (40.5)
61–75	USA, Japan, Israel, China	156	156	69.0 (65.0–73.0)	71 (45.5)	14.1 (0.4–14.1)	110 (70.5)
>75	USA, Japan, Israel, China	130	130	80.0 (78.0–84.0)	56 (43.1)	20.7 (0.7–20.7)	103 (79.3)
intended case scenario							
RBDB -test2	Israel	1825	1676	67.0 (51.0–77.0)	801 (43.9)	24.0 (22.0–25.2)	188 (10.3)

Train set, internal test set from UVAF-test, external test sets (RBDB-test, SHDB-test and CPSC-test), sex and age test sets and the intended case scenario test set RBDB-test2. Median (***μ***) and the interquartile range (Q1–Q3) are reported for age and recording length. The University of Virginia dataset (UVAF) from the USA, the Saitama Hospital dataset (SHDB) from Japan, the China Physiological Signal Challenge (CPSC) from China and Rambam Hospital dataset (RBDB) from Israel. Sex was not reported for a single patient in CPSC-test and thus we excluded this recording from the sex group. Female and AF prevalence percentages (%) are calculated out of total number of recordings.

### Deep learning

**Fig. 2:**
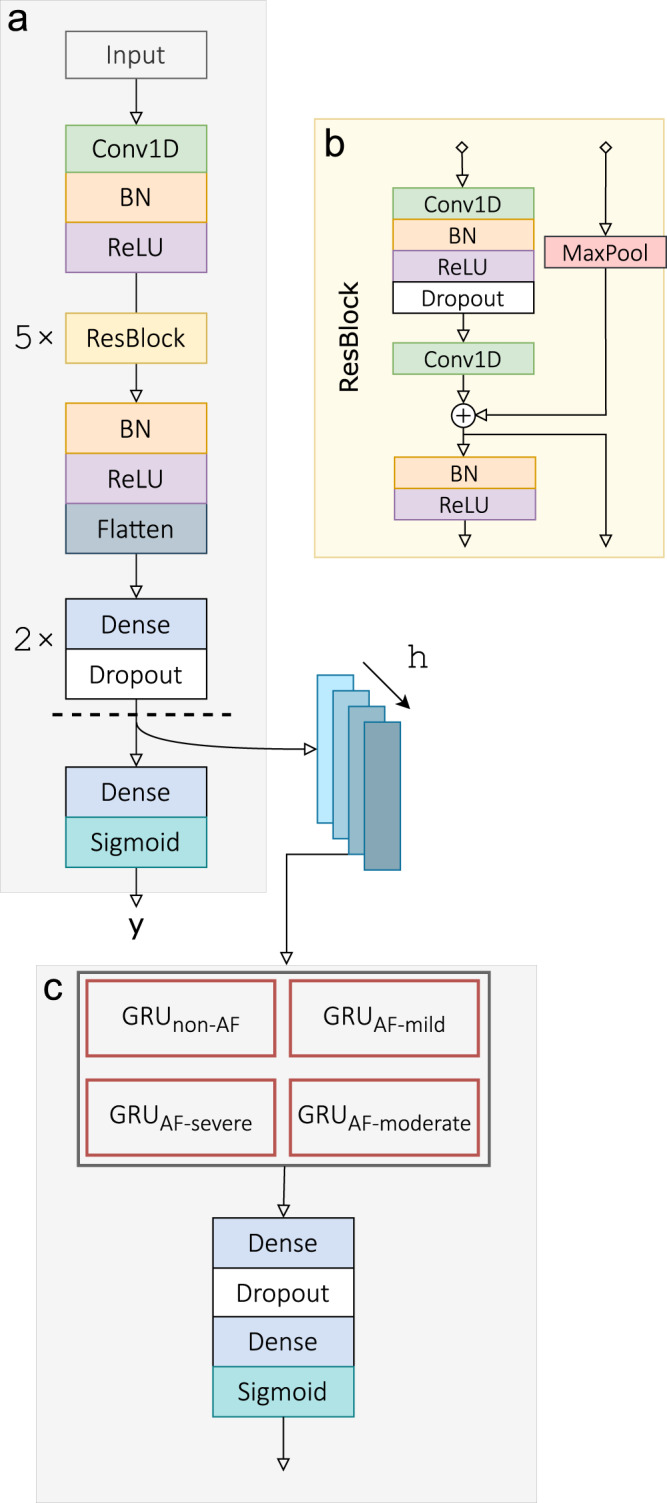
ArNet2 architecture. A deep CNN with ResNet blocks (ResBlock) is trained at the first stage, (**a**). The ResBlock architecture is given in (**b**). This first stage network is trained for the task of binary classification on 60-beat random windows. Before the last layer, the embedded features are extracted. The second stage consists of an RNN with GRU units (**c**). Its inputs are the extracted embedded features from the previously trained CNN, both from the current window and its h preceding windows (when available), all being temporally concatenated. Depending on the AF_l_ severity label predicted by the CNN, a GRU layer is chosen from the pool, which takes as input the window to predict as well as h previous ones. It then goes through two dense layers and outputs a probability label of AF_l_ for each window.

ArNet2 builds on our previous work developing ArNet^9^. The input is a 60-beat RR-interval window (w_s_), i.e., 59 RR intervals, and the output is a binary classification of AF_l_/non-AF_l_. The DL architecture is divided into two parts (Fig. 2): a first part constituted of residual blocks and used to build representation of an ECG signal window, thereby creating an embedding. The second part consists of a sequence encoder made of GRUs to take the time representation into account. ArNet2 was benchmarked against ArNet and a gradient boosting (XGB) model taking as input heart rate variability (HRV) features^9^. The following statistics were computed to assess model performance for the individual 60-beat-window classification task: sensitivity (Se), specificity (Sp), positive predictive value (PPV), negative predictive value (NPV), AUROC, area under the Precision-Recall Curve (AUCPR) and the harmonic mean between Se and PPV (F1). In addition, the absolute difference between the estimated and reference AF Burden (AFB) denoted $\left| {{{{\mathrm{E}}}}_{{{{\mathrm{AF}}}}}\left( \% \right)} \right|$ was computed as an overall clinical performance measure.

### Overall model performance

**Fig. 3:**
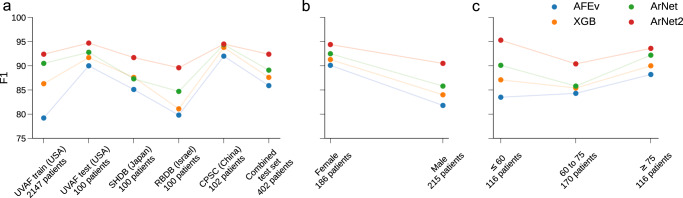
F1 performance for the machine learning models across different distribution shifts. Performance for XGB, ArNet, ArNet2 and a rule-based approach AFEv^17^ in classifying AF_l_ events across geography (**a**), sex (**b**) and age (**c**).

The best UVAF-test set score for window classification was obtained for ArNet2 (Fig. 3a and Supplementary Table 1) with an F1 of 0.95 compared to F1 of 0.93 for ArNet and 0.92 for XGB. All machine learning methods were superior to the AFEv rule-based approach (F1 = 0.90, $\left| {{{{\mathrm{E}}}}_{{{{\mathrm{AF}}}}}\left( \% \right)} \right|$ = 3.21 (1.28–10.72)). The $\left| {{{{\mathrm{E}}}}_{{{{\mathrm{AF}}}}}\left( \% \right)} \right|$, for the UVAF-test set was 0.1 (0.0–2.41) for ArNet2, 0.08 (0.0–3.29) for ArNet and 1.42 (0.3–5.31) for XGB. Supplementary Fig. 1 shows the histogram of $\left| {{{{\mathrm{E}}}}_{{{{\mathrm{AF}}}}}\left( \% \right)} \right|$ for each AF_l_ severity level for ArNet2. A proportional T-test revealed that the F1 performance was significant between ArNet2 and ArNet (*p* < 0.001). A paired T-test revealed that the gap in $\left| {{{{\mathrm{E}}}}_{{{{\mathrm{AF}}}}}\left( \% \right)} \right|$ was significant (*p* < 0.001) between ArNet2 and ArNet.

### Performance across geography

Table 2 and Fig. 3a present performance statistic for ArNet2 across different geography groups. When considering F1, ArNet2 consistently outperformed other DL models (Supplementary Table 1 and Fig. 3a) with all external test sets apart for the CPSC-test set, where ArNet2 scored a F1 of 0.95 compared to F1 of 0.95 for ArNet and 0.94 for XGB. The range of ArNet2 performance across the different geography groups varied in the range of F1 = 0.90–0.95, with the best performance obtained for the CPSC-test set and the lowest for the RBDB-test set. The median performance in terms of $\left| {{{{\mathrm{E}}}}_{{{{\mathrm{AF}}}}}\left( \% \right)} \right|$ was similar between ArNet2 and ArNet, but the Q3 was systematically smaller for ArNet2, Supplementary Table 2.

**Table 2:** Performance measures of ArNet2 across geography, sex and ages.

	F_1_	AUROC	AUCPR	Se	Sp	PPV	NPV	$\left| {{{{\mathrm{E}}}}_{{{{\mathrm{AF}}}}}\left( \% \right)} \right|$ μ(Q_1_-Q_3_)
Geography								
UVAF-test	0.95	0.99	0.99	0.93	0.96	0.96	0.93	0.10 (0.0–2.41)
SHDB-test	0.92	0.99	0.93	0.89	0.98	0.94	0.96	0.59 (0.10–3.23)
RBDB-test	0.90	0.99	0.98	0.83	0.98	0.97	0.86	0.21 (0.09–7.59)
CPSC-test	0.95	0.99	0.98	0.95	0.97	0.94	0.97	0.0 (0.0–6.02)
Combined test set	0.92	0.99	0.98	0.89	0.97	0.96	0.93	0.32 (0.01–3.62)
Sex								
Female	0.94	0.99	0.99	0.92	0.98	0.97	0.95	0.14 (0.0–2.46)
Male	0.90	0.98	0.97	0.86	0.97	0.95	0.91	0.71 (0.06–4.79)
Ages								
≤60	0.95	0.99	0.99	0.93	0.99	0.97	0.98	0.14 (0.0–2.81)
61 to 75	0.90	0.98	0.97	0.86	0.97	0.96	0.90	0.47 (0.05–5.15)
>75	0.94	0.98	0.99	0.91	0.94	0.96	0.88	0.22 (0.0–2.66)

For each performance measure the highest entry is underlined for the per sex and per ages statistics.

### Performance across sexes

Table 2 and Fig. 3b present results for ArNet2 across different sex groups. ArNet2 performed significantly better (*p* < 0.001) for female (F1 = 0.94) compared to male (F1 = 0.90) windows. The ArNet2 $\left| {{{{\mathrm{E}}}}_{{{{\mathrm{AF}}}}}\left( \% \right)} \right|$ for the female group was 0.14 (0.0–2.46) compared to 0.71 (0.06–4.79) for the male group, Supplementary Table 3. This relative trend was also observed for the other models (XGB and ArNet) as well (Supplementary Table 4 and Fig. 3b).

### Performance across age groups

Table 2 and Fig. 3c present performance measures for ArNet2 across different age groups. ArNet2 performed better for adult patients aged ≤60 years (F1 of 0.95) compared to patients >60 years (F1 of 0.90 for ages 61–75 years and 0.94 for >75 years). Similarly, the $\left| {{{{\mathrm{E}}}}_{{{{\mathrm{AF}}}}}\left( \% \right)} \right|$ for the ≤60 years age group was smaller (0.14 (0.0–2.81)) than for the older age groups (0.47 (0.05–5.15)) for 61–75 years and (0.22 (0.0–2.66)) for >75 years, Supplementary Table 5. Similar performance was measured for the other models (XGB and ArNet) as well (Supplementary Table 6 and Fig. 3c).

### Performance for simulating intended use scenario

**Fig. 4:**
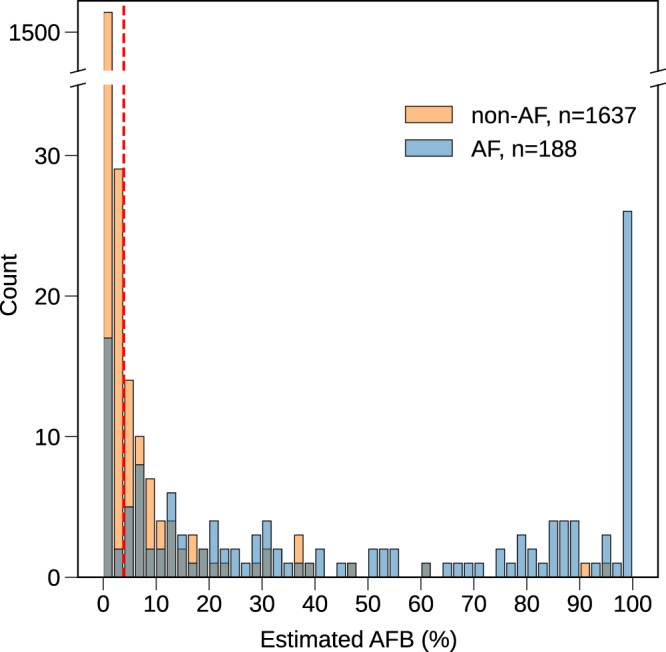
Histogram of the ArNet2 estimated AFB (AF Burden) for the RBDB-test2. The RBDB-test2 dataset (*n* = 1825) simulating the intended use scenario. The red dashed line is drawn at AFB = 4%.

In the simulated intended use scenario, we consider a fully automated AF diagnosis algorithm based on ArNet2 at the patient level and while setting a decision threshold based on the AFB at 4% (Fig. 4). ArNet2 yielded a PPV of 0.74, NPV of 0.99, Se of 0.90, Sp of 0.96, F1 of 0.81, AUCPR of 0.90 and AUROC of 0.96 in performing a fully automated diagnosis at the patient level (see Table 3). ArNet2 performed better for the female group for all performance measures compared to the male group (see Table 3). The AUROC, Se, Sp and NPV were higher for adult patients aged ≤60 years versus the older groups >60 years. The PPV, F1 and AUCPR however, were higher for the older groups. Finally, XGB misclassified 16% (30/188) and ArNet2 misclassified 10% (19/188) of the positive AF recordings while the specificity was close for the two algorithms (97% and 96% respectively).

**Table 3:** Performance measures of ArNet2 for the intended use scenario and across sex and ages at the patient level.

	F_1_	AUROC	AUCPR	Se	Sp	PPV	NPV
RBDB-test2	0.81	0.96	0.90	0.90	0.96	0.74	0.99
Sex							
Female	0.86	0.96	0.92	0.92	0.97	0.82	0.99
Male	0.77	0.95	0.88	0.89	0.96	0.67	0.99
Ages							
≤60	0.72	0.99	0.90	0.94	0.98	0.59	1.0
61–75	0.80	0.95	0.89	0.86	0.97	0.75	0.98
>75	0.83	0.95	0.92	0.91	0.93	0.77	0.98

AFB threshold is defined at 4%. For each performance measure the highest entry is underlined for the per sex and per ages statistics.

### Error analysis

#### Combined test set error analysis

**Fig. 5:**
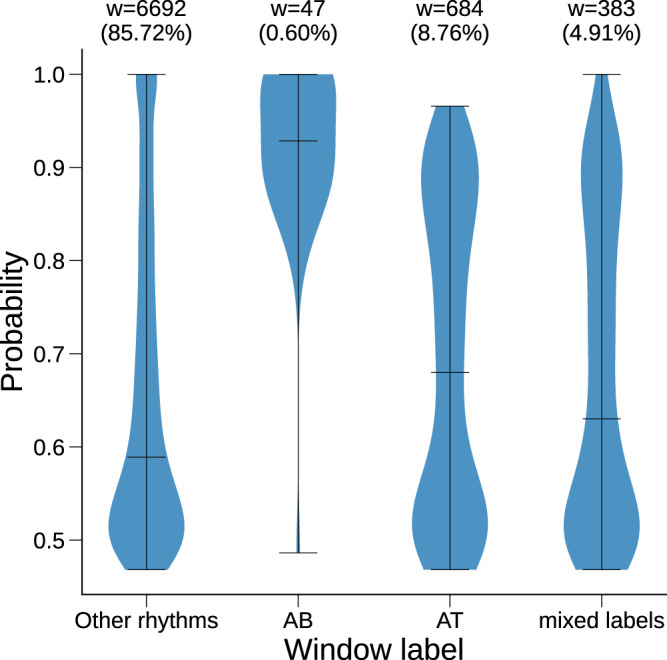
The probability output of ArNet2 for the combined test set FP windows grouped to the true window labels. The represented rhythms shown in the violin plots are Other rhythms, Atrial Bigeminy (AB), Atrial Tachycardia (AT) and mixed labels. The mixed labels group consists of windows with AF_l_ and other dominant rhythm so that the label of this window is non- AF_l_. w refers to the total number of FP windows in each violin plot while the proportion of FP windows for a given rhythm over the total number of FP windows is written in parenthesis.

Out of 300 combined manually annotated test set recordings, 209 patients had AF events and no AFL events, 5 patients had AFL events and no AF events and 14 had a mixture of AF and AFL events. In total, 69.68% of the AFL windows were misclassified as non-AF_*l*_ by ArNet2. This represented 47.72% of all False Negatives (FN) and reflects the fact that ArNet2 performs poorly in detecting AFL events. This led to underestimation of the $\left| {{{{\mathrm{E}}}}_{{{{\mathrm{AF}}}}}\left( \% \right)} \right|$ which may result in the misclassification of these patients as non-AF_*l*_. In parallel, 85.72% of the FP windows were labeled as other rhythms, 8.76% as atrial tachycardia (AT), 4.91% as mixed labels and 0.6% as atrial bigeminy (AB) (see Fig. 5). Examples of misclassified windows are shown in Supplementary Fig. 2.

#### Simulating intended use scenario error analysis

Of the FP patients, 65.0% were diagnosed with other coronary artery diseases (CAD), including myocardial infarction and congestive heart failure. For the FN cases, the estimated cumulated duration of AF_*l*_ events were very low with 64.43, (39.78–78.55) sec. The AFB distribution is shown in Fig. 4. A total of 59 FP cases were reviewed by a cardiologist; the most prevalent arrhythmias that were misclassified as AF_*l*_ were AT (40.67%), premature ventricular contraction (25.42%) and sinus arrhythmia (23.72%).

## Discussion

The main contribution of the research are the methods and results to assess the generalization performance of a new DL algorithm for AF events detection across different geography groups, sexes and ages. Generalization performance was evaluated using four test sets from different countries (USA, Japan, Israel and China). Two of those the test sets (Israel and Japan) were newly elaborated for this research. ArNet2 generalization performance across geography groups was consistently higher than that of the benchmark algorithms (Fig. 3a). ArNet2 performance across geography groups varied from an F1 of 0.90 for RBDB to 0.95 for CPSC. Min-max difference in performance across all geography test-sets was calculated for the four models. The lowest difference was obtained for ArNet2 with 5.7% while it was 9.7% for ArNet, 12.7% for XGB and 12.8% for AFEv. The lower Min-max difference in ArNet2 while achieving the highest performance for all external test sets highlights the better generalization performance for ArNet2. Although training a DL model is time and resource consuming its inference time can be short. In our context, the average inference time for a window classification was 0.04 ms for ArNet2 versus 0.54 ms for XGB. Thus, in addition to the performance gain of ArNet2 over XGB we also report on a gain in running time. Previous research have shown interesting results in AF classification from beat-to-beat interval^13,14^. However, we could not objectively benchmark their models against ArNet2 since their codes are not open source and the test databases they report on differ from our test databases. AFL events were identified in 2.94% of the UVAF, 7.22% of the SHDB, 14.13% of the RBDB and 0.43% of the CPSC recordings. When assessing ArNet2 across sexes, performance was higher for females than males. The vast majority (83.34%) of AFL windows in the combined test set belonged to the male group and only 16.66% belonged to the female group. Thus, the prevalence of AFL among men was significantly higher than in women in our test set. When assessing ArNet2 for different age groups, the percentage of AFL windows out of the total AF_l_ windows per age group was found to be 0.8% for adults aged ≤60 years, 10.5% for adults aged 61–75 years and 6.3% for the >75 age group. Taken together, the relative prevalence of AFL to AF was significantly higher in the elderly population (>60 years). The observations made for ArNet2 performance across geography, sexes and age groups were consistent for XGB and ArNet (Fig. 3).

The observations on performance presented by patient sex are consistent with a previous single-center study conducted by a group from Wisconsin (USA)^15^, who analyzed medical records from a dataset of 58,820 patients. The authors reported that AFL was 2.5 times more common in men (*p* < 0.001). The overall incidence of new cases of AFL during the four-year study period was 88 per 100,000 person-years, ranging between 5 per 100,000 in patients aged <50 years and 587 per 100,000 in patients >80 years of age. Since models working from beat-to-beat intervals can hardly detect AFL windows, we suggest that the higher prevalence of AFL windows found in RBDB (versus other geography groups), male (versus female) and aged (versus younger) groups explains the lower performance of ArNet2 for these subpopulations. These observations may have important clinical implications suggesting, for example, that for the male population, detection of AF_l_ events may necessitate the use of a portable device measuring continuously the raw ECG waveform. However, there is limited research comparing AF_l_ events detection based on beat-to-beat intervals versus on raw ECG data and thus it remains to be experimentally proven that analyzing the raw ECG versus the beat-to-beat interval variation would yield better results. Taken together, epidemiological consideration on the relative prevalence of AFL to AF across different geography groups and across age groups is warranted to better define the population sample expected to benefit from data-driven models based on the beat-to-beat interval variation versus the raw ECG waveform. In terms of median $\left| {{{{\mathrm{E}}}}_{{{{\mathrm{AF}}}}}\left( \% \right)} \right|$, ArNet2 better performed on non-AF_l_ and AF_sev_ patients then for other AF_l_ severity levels for all datasets, Supplementary Table 7.

The second main contribution of this research is the elaboration of ArNet2, a new robust DL model for AF_l_ events detection from long term beat-to-beat interval time series. In the context of AF diagnosis based on the irregularity of the RR-interval, notable research include single features drawing from information theory such as the coefficient of sample entropy (CosEn)^16^ or Lorenz-based features^17^. We have shown the superiority of ArNet2 over classical machine learning approaches taking engineered features such as CosEn as input^9^. ArNet2 also significantly outperformed (*p* < 0.001) ArNet (Fig. 3 and Supplementary Table 1, Supplementary Table 4 and Supplementary Table 6).

The third main contribution of this research was the evaluation of ArNet2 on a large dataset of 1,825 consecutive recordings collected from Rambam Hospital Holter clinic. While considering an AFB decision threshold at 4%, ArNet2 yielded better performance for female than male patients for all performance measures. For the age groups, the AUROC, Se, Sp and NPV were higher for the adults aged ≤60 years than the older >60 years groups while the PPV, F1 and AUPRC were higher for the older groups. This is due to the very low prevalence of AF_l_ recordings in the ≤60 years group (2.64%) versus in the older >60 years groups (14.89%). Among the 1,637 non-AF_l_ recordings, a total of 59 would have been flagged as AF_l_, subsequently requiring human review of the respective ECG recordings and thus incurring some cost. While the intention of the automated data driven analysis is to achieve the highest PPV possible, among these 59 recordings, 50 had another cardiac abnormality and thus review of their ECG by a qualified health professional would have been worthwhile anyway. This further highlights the perspective of our robust data-driven algorithm. Among the 188 patients with AF_l_, a total of 169 were correctly diagnosed with AF_l_ in a fully automated manner. When comparing ArNet2 on a real case scenario versus benchmarked algorithms, the results showed that the performance of ArNet2 led to a significantly higher number of AF_l_ individuals being correctly diagnosed versus XGB. For the FN cases, the cumulated duration of AF_l_ events were very short with 64.43, (39.78–78.55) sec. Thus, although these individuals had AF_l_ events it is not certain that they would have been treated for AF given the short episodes. In addition, ArNet2 accurately estimated the AFB which may be an important statistic when considering treatment options and dosage although it is not included in current management guidelines.

This research had several limitations. First, we had at our disposal a large training set with UVAF-train. However, the recordings annotations were originally generated with the Philips Holter software and only 53% of these annotations were reviewed as discussed in Carrara et al.^7^ This is an intrinsic limitation of our model training higher performance may be reached using improved annotations on the UVAF-train set. Second, for the window classification experiments, the prevalence of AF for the test sets were higher than that typically encountered clinically (See Table 1, “AF prevalence”) and thus prevalence-dependent performance measures (e.g., F1, PPV) will likely be lower when applied prospectively in real-world populations. In addition, the decision threshold of ArNet2 was optimized on the validation set to maximize the F1 measure. Yet, since the F1 measure is prevalence-based this may be a limitation in the case of a non-representative validation set. Third, generalization was evaluated on a limited number of Holter recordings (402 overall) and for a limited number of geographies groups (four different countries) without elaboration on racial diversity. Although the performance of ArNet2 was high and outperformed benchmark algorithms, it failed to detect a significant proportion of AFL windows (69.68%), resulting in variation in performance across geography, ages and sexes. This is an intrinsic limitation of a data-driven algorithm working on beat-to-beat interval variation versus raw ECG data. Yet on the other hand the beat-to-beat interval time series present some advantages over using the raw ECG: (1) it can be measured on multiple pulsatile signals measured using wearable sensors (i.e. not only the ECG), (2) the R-peak is a robust fiducial point on the ECG and thus less prone to noise than using the raw ECG waveform and (3) there is a significant different in the computational time processing a raw ECG typically sampled at >200 Hz versus a beat-to-beat interval time series. Overall, a rigorous and quantitative comparison of DL methods using the raw ECG versus the beat-to-beat time series as input is wanted. When detecting AF_l_ events, 8.76% of FPs were labeled as AT rhythms. This is an intrinsic limitation of working with Holter recordings for which the ability of the cardiologist to distinguish between AT and AFL with 2:1 AV conduction is extremely challenging without performing addition maneuver (vagal maneuver or/and adenosine test). Another 4.91% of FPs were mixed-label but with a non-AF_l_ dominant rhythm. This is also an intrinsic limitation of the methodology, since we work using 60-beat windows. In addition, 85.72% of FPs were labeled as other rhythms. More work is needed to understand the reasons for misclassification of these windows. Finally, a panel of annotators would have been preferable to strengthen the precision of the reference annotations even further for our test sets. Previous work using such methodology has mostly done so for short ECG recordings, whether 12-lead ECG of typically 10 seconds long or short (few seconds long) single ECG. Indeed, with our methodology it took on average 45 min for the fellow in cardiology to complete the review of a single Holter recording.

In the present work, and similar to previous research on AF detection using machine learning, AF and AFL events were grouped into a single AF_l_ class. However, distinguishing between AF and AFL has clinical impact on the diagnosis and management of patients with one or the combination of these atrial arrhythmias. Unlike AF, in which rhythm control and rate control are reasonable strategies, maintenance of sinus rhythm is desirable in most patients with AFL to control symptoms. For most AFL patients, radiofrequency catheter ablation is preferred over pharmacologic therapy because of the high success rate and low rate of complications. In addition, many patients with AFL have episodes of AF. Simultaneous ablation of AF and cavotricuspid isthmus (CTI)-dependent AFL can be performed when both arrythmias are recorded before the procedure. However, the best approach for patients referred for ablation with CTI-dependent AFL, without history of AF has not been defined^18,19^. In conclusion, because AF and AFL are managed differently, with the exception of anticoagulation therapy, a significant proportion of patients would benefit from the diagnostic tools distinguishing between the two conditions. Although the incidence of AFL in the general population is still uncertain, the analysis of Granada et al.^15^ estimated it to be 200,000 new cases per year in the United States. There is thus a strong motivation to create data-driven algorithms that can detect and distinguish between AFL and AF events. For this purpose, the development of data-driven models based on raw ECG data may be necessary.

Overall, the datasets for our analysis totaled 4298 recordings and over 99,705 h of continuous data. We demonstrated that our new model is robust and generalizable across geography, ages and sexes being consistently significantly superior to benchmarks. We attributed differences in performance across geography groups, sex and age to the relative prevalence of AFL. However, other factors such as the prevalence of ectopic beats may also play a role in the misclassification of some windows. Since AF and AFL have different clinical management pathway, our findings support the need to discriminate between the AF and AFL classes in future work. Research is also warranted in documenting the relative prevalence of AFL to AF according to age as well as across geography. The algorithm developed can be used to power remote monitoring wearable solutions such as smartwatches or single lead ECG patches. This robust data-driven capability imbedded in wearables opens to the perspective of long term continuous remote monitoring for diagnosis of AF in groups such as those with embolic stroke of undetermined source or to follow-up on ablation therapy in those at risk for recurrent AF.

## Methods

We divided our experiments in two steps. First, we aimed at creating a robust model, denoted ArNet2, for classifying individual 60-beat windows as AF_l_ or non-AF_l_. In order to assess the performance of ArNet2, we created test sets from different geography origin for which the Holters were annotated at the beat level. Given the new ArNet2 model, we evaluated the performance of ArNet2 in identifying AF_l_ individuals, that is a classification task at the patient level. This was performed on a larger and more representative test set for which we had AF_l_ diagnosis labels at the patient level.

### Development set

The UVAF^7,8^ consists of ECG Holter recordings of patients for whom the University of Virginia health system physicians ordered Holter monitoring between December 2004 to October 2010. Indications for the Holter recordings included palpitations (40%) or syncope and dizziness (12%). The dataset contains 2247 annotated recordings of individual patients over the age of 18 years. Beat labels were automatically generated by the medical monitor Philips Holter software. Part of these recordings (52.3%) were manually overread for the presence of AF_l_ as described in Carrara et al.^7^ Recordings were recorded using Philips Holter software and digitized at 200 Hz. UVAF was used to train (UVAF-train, *n* = 2147) and evaluate (UVAF-test, *n* = 100) the DL models.

### Test sets

The demographic characteristics of patients from the test sets are summarized in Table 1 and Supplementary Fig. 3. Age and sex were not statistically different across the test sets using the Kruskal-Wallis statistical test with Dunn’s post hoc analysis. The age was statically different (*p* < 0.001) between the training and test sets. We elaborated three test sets denoted UVAF-test, RBDB-test and SHDB-test. These include two newly created datasets: the Rambam Hospital dataset (RBDB) from Israel and the Saitama Hospital dataset (SHDB), Japan. In compliance with all relevant ethical regulations we obtained for the newly collected datasets, ethical approval for using the retrospective de-identified data was granted by the Saitama Medical University institutional ethics committee under IRB number 20–030 and the Rambam Health Care Campus institutional ethics committee under IRB: D-0402-21. RBDB and SHDB consisted of the raw ECG recordings and corresponding medical report prepared following the Holter examinations. The reference rhythm annotations at the beat level were obtained for a subset of these datasets in order to create RBDB-test and SHDB-test. For that purpose, a total of 100 recordings were selected from the original databases while stratifying by age, sex and diagnosis for AF_l_. Specifically, 80 recordings from patients that had AF_l_ based on the cardiology report were selected for each test set. For the RBDB-test and SHDB-test, the AF_l_ diagnoses were obtained from the medical report prepared following the patient’s examination. As no patient reports were available for UVAF, the diagnosis was inferred from the AF_l_ events annotated in the recordings. For the CPSC-test the complete available open dataset was used as is. The CPSC-test^20,21^ contains 1,136 variable-length ECG recordings extracted from lead I and lead II of 102 long-term dynamic ECGs, digitized at 200 Hz. The variable-length ECG recordings fragments were analyzed individually and then, in order to report the performance measures per patient, the classification outputs were grouped into patient recordings using the ID numbers located in the filename. The RBDB-test consists of 100 ECG recordings of adults for whom physicians from multiple hospital departments ordered Holter monitoring between October 2013 and November 2021. Holter recordings from the RBDB-test were recorded using PathFinder Holter monitor and digitized at 128 Hz, with 12-bit resolution over a 10 mV range. The SHDB-test consists of 100 adult ECG recordings of patients for whom treating physician ordered Holter monitoring between November 2019 and January 2022. Holters from the SHDB-test were recorded using Fukuda Holter monitor and digitized at 125 Hz and two leads were recorded, modified CC5 and NASA leads. The UVAF-test, RBDB-test and SHDB-test recordings were manually re-annotated (see Expert annotations of test sets). For the CPSC-test AF and AFL beat labels were created automatically by the Holter software and later reviewed by cardiologists to correct the onsets and offsets of AF_l_. The open access labels were used as provided. At last, all test sets were combined under Combined test set to assess global performance. Based on the clinical data at our disposal and Holter findings we estimate that most AFL cases were typical. Apart from geographic groups, generalizability performance was assessed with respect to sex and age groups. Performance across sex was assessed for the combined test for each sex group. Performance across ages was assessed for the combined test set for each age group; adults ≤60 years, adults aged 61–75 years and adults > 75 age group.

### Simulating intended use scenario

In addition to the RBDB-test recordings, an additional 1725 consecutive recordings retrospectively obtained from the clinic from the period August 2017 and November 2021, were obtained from the Rambam Hospital Holter clinic. We combined those additional recordings with RBDB-test and denoted the new dataset RBDB-test2. The free texts of the Holter examination reports were reviewed manually for the diagnosis of AF and AFL. If short AF or AFL events were explicitly reported, then the corresponding Holters were reviewed by a fellow in cardiology (AE) and if the event(s) were shorter than 30 s, then a non-AF_l_ label was given to the recording. If at least one event was longer than 30 s, then an AF_l_-positive label was given to the recording^22^. As a result, the RBDB-test2 set consisted of 1825 recordings with a binary label for the presence or absence of AF_l_ rhythm. This subset of RBDB was used to simulate the results of the new algorithm in performing a fully automated diagnosis on an intended use population sample.

### Expert annotations of test sets

The Holter ECG makes it possible to record the cardiac electrical activity continuously over 24 h or longer. It helps physicians to detect arrhythmic disorders. In annotating AF events for this work the heart rate (HR) time series, RR time interval variability and QRS complexes were mainly used^23,24^. The HR time series enables to assess whether the HR is normal defined as within the interval of 60 to 100 bpm or in tachycardia defined as an HR over 100 bpm. When a tachycardia was detected, it was determined if this was sinus rhythm or not and the type of tachycardia was annotated. Yet, some AF events happen during periods of normal or low HR. This can be the case in patients receiving treatments lowering HR (betablockers). AF is a rhythm disorder presenting irregular variation in the RR time series. This irregularity enables to detect AF events even at a normal HR. Irregularities of the RR time series may also be due to the detection of atrial, junctional and ventricular extrasystoles. However, these extrasystoles are easily recognizable and do not represent a diagnostic challenge. Thus, it is possible to effectively detect AF events occurring during normal HR. AFL is defined as a disorder of regular supraventricular rhythm due to a macro reentry in right atrium, recognized with the presence of F-wave on ECG. The distinction between AF and AFL is generally easy. However, in the event of an AFL with a HR faster than 150 bpm, the F-wave are often not visible, and it can pose a diagnostic challenge. In hospital, the vagal maneuver or the adenosine test are performed in order to slow down the HR to better see the atrial activity and distinguish the F-wave. However, this is infeasible in working with retrospective recordings. The distinction between AF and AFL at a high HR thus remains a limitation. In supraventricular rhythm disorder QRS complexes are less than 120 ms. However, in some instances, patients can present wide QRS, when a bundle branch is present. It can be permanent (organic bundle branch) or occurring only for high HR (functional bundle branch block). This may mimic ventricular tachycardia, characterized by wide QRS tachycardia. However, in AF the rhythm is irregular which enables a discriminative diagnosis between AF and ventricular tachycardia in these instances Using this protocol, supraventricular arrhythmias were annotated at the beat level with no over-reading of automated labels into four categories: (1) AF; (2) AFL; (3) atrial tachycardia (AT); (4) Other supraventricular tachycardias such as Wolf-Parkinson-White and intranodal tachycardias and (5) other such as NSR that where not labeled. No semi-automated analysis or technician was used to pre-identify AF events. Rather the fellow worked from the raw Holter recording with information on instantaneous HR and RR interval variation being pre-computed by the software.

### Preprocessing

All datasets were resampled to 200 Hz using an anti-aliasing filter. A zero phase second-order infinite impulse response bandpass filter with the passband [0.67–100]Hz^25^ was used to remove baseline wander and high frequency noise. Beat annotations were detected using *epltd* implementation of the Pan and Tompkins algorithm^26^. The RR intervals, based on the beat annotations, were divided into non-overlapping windows of 60-beats (Fig. 1). For each window, the bSQI index^27^ was computed with an agreement window of 50 ms. We used *epltd* as the reference peak detector and xqrs^20^ as test peak detector. Regarding the UVAF, some reference beat labels were missing. To account for that, windows with over 10 s of missing beat labels were excluded. Patients presenting over 25% of 60-beat windows with missing beat labels based on this criterion were discarded. Among the remaining recordings, windows with bSQI lower than 0.8 were considered of low quality and were excluded. Recordings with a rate of exclusion, i.e. the number of excluded windows over the total number of windows exceed 75% were considered as corrupted by noise and were discarded.

### Deep learning for AF events detection

#### ArNet2 architecture

The first part is formed by stacking n_b_ of 5 residual blocks that consists of two 1D convolutional layers per block, with a Batch Normalization (BN) layer and Rectified Linear Unit (ReLU) adopted from He et al.^28^ prior to each convolutional layer. A dropout layer with rate (d_r1_) of 0.2 is added between the blocks with a shortcut connection and Max Pooling. Every two blocks, the number of filters from one layer to the next doubles while the input length is divided by two, with initial number of filters (n_f_) of 64 and a filter length (f_l_) of 10. The final block is composed of Flattening and three successive Dense and Dropout layers with dropout rate (d_r2_) of 0.5, at this point, the embedded features are extracted. The initial number of neurons of first dense layer, following the ResNet layers (n_d_) is 512, being divided by two each time. After the extraction, a last Dense layer of size 1 outputs a binary classification for each input window. The chosen loss for the first training step is a weighted binary cross-entropy loss, giving higher importance to the windows labeled as AF_l_, and optimized with Adam^29^ with a learning rate *α* of 10^−2^. Then, in the second step, a Recurrent Neural Network (RNN) takes as input the embedded features as well as the corresponding classification output from the previous step. This RNN consists of 4 gated recurrent units (GRU) with the same architecture, two dense layers and a sigmoid function which acts as an activation function and outputs a probability label of AF_l_ for each window. Each GRU is trained with windows belonging to a target population (Non-AF_l_, AF_mild_, AF_mod_ and AF_sev_) to better capture the temporal distribution of AF_l_ events for a given AFB corresponding to a given severity level, as defined in Chocron et al.^9^. For each window, the embedded features from the 6 preceding windows (when available, h) are concatenated along the time axis. Our intended use scenario is directed towards long-term monitoring of cardiac activity. Thus, we assume the length of the recordings to be several hours long. To initialize the RNN unit on the first few windows a total of h hidden state will be initialized as a matrix of zeros. Then, one GRU unit is selected out of the four based on the patient’s AFB and fed with the resulting features array. The output of the model based on the GRU unit is a classification of label per window. Considering the RNN units, the average recording time processed per window classification was about eight minutes. During training, the AFB used is the true AFB. However, during inference, the estimated AFB based on the first step output classification is used.

#### Training strategy

For each window, a label, i.e. AF_l_ or Non-AF_l_ was assigned, based on the most prevalent beat label in the 60-beat window. DL models were trained for binary classification of individual windows as AF_l_ or non-AF_l_. Hyperparameters were optimized and selected using Bayesian search^30^ using 5-fold cross-validation. We used early stopping strategy on the validation loss with minimum change of 0.001 to avoid overfitting. The hyperparameters search space is described in Supplementary Table 8. The proposed network ArNet2 was benchmarked against a gradient boosting (XGB) model and ArNet developed in our previous work^9^, We also added the comparison to the AFEv^17^ rule-based approach for comparison against machine learning models. The models were optimized to maximize the area under the receiver operating characteristic (AUROC) on the validation set and the threshold on the output probabilities was defined as the point which maximized the F1 on the training set. Models were trained and optimized on the UVAF-train and evaluated on the internal test set (UVAF-test) as well as the external test sets (RBDB-test, SHDB-test, CPSC-test). Models were built using TensorFlow 2.4 and trained using a single NVIDIA A100 GPU.

### Performance statistics

Performance was evaluated at the window (60-beat) level and at the level of the overall recording in estimating the AFB. The following statistics were computed to assess model performance for the individual 60-beat-window classification task: sensitivity (Se), specificity (Sp), positive predictive value (PPV), negative predictive value (NPV), AUROC, area under Precision-Recall Curve (AUCPR) and the harmonic mean between Se and PPV (F1).

A clinical performance measure, namely the AF Burden (AFB), which we defined as the percentage of time spent in AF_l_. Research has suggested superior prognostic value of AFB for stroke^31^ compared to a binary diagnosis. Patients were categorized into four groups based on their AFB as defined in our previous work^9^: Non-AF_l_: <30 s total time spent in AF_l_^14^, Mild AF (AF_mild_): >30 s total time spent in AF_l_ and AFB is <4%^22,32^, Moderate AF (AF_mod_): AFB is in the range 4–80%, Severe AF_l_ (AF_sev_): AFB > 80%. The AFB was defined in below equation^9^.

1 $$AFB = \frac{{\mathop {\sum }\nolimits_{n = 1}^N t_i \times {\Bbb I}_i}}{{\mathop {\sum }\nolimits_{n = 1}^N t_i}}$$

with **N** representing the number of available windows, **t**_**i**_ the length of the **i**_**th**_ window (ms) and ${\Bbb I}_{{{\mathbf{i}}}}$ the unity operator which was equal to 1 for **AF**_**l**_ and otherwise zero.

For a given recording, E_AF_ (%) was defined in below equation.

2 $${{{\mathrm{E}}}}_{{{{\mathrm{AF}}}}}({{{\mathrm{\% }}}}) = \frac{{\mathop {\sum }\nolimits_{{{{\mathrm{n}}}} = 1}^{{{\mathrm{N}}}} {{{\mathrm{t}}}}_{{{\mathrm{i}}}} \times \left( {\widehat {{{{\mathrm{y}}}}_{{{\mathrm{i}}}}} - {{{\mathrm{y}}}}_{{{\mathrm{i}}}}} \right)}}{{\mathop {\sum }\nolimits_{{{{\mathrm{n}}}} = 1}^{{{\mathrm{N}}}} {{{\mathrm{t}}}}_{{{\mathrm{i}}}}}}$$

where y_i_ is a binary value representing the window label and $\widehat {{{{\mathrm{y}}}}_{{{\mathrm{i}}}}}$ is the binary label predicted by the model. The binary value 1 was used for AF_l_ and 0 for Non-AF_l_. The $\left| {{{{\mathrm{E}}}}_{{{{\mathrm{AF}}}}}\left( \% \right)} \right|$ was computed for each AF_l_ target population (Non-AF_l_, AF_mild_, AF_mod_, AF_sev_). The median and interquartiles, as measures of central tendency and dispersion, were reported for $\left| {{{{\mathrm{E}}}}_{{{{\mathrm{AF}}}}}\left( \% \right)} \right|$.

For the simulated intended use scenario, the F1, AUROC, AUPRC, Se, Sp, PPV and NPV performance statistics were calculated for the classification task at the patient level. In order to set a decision threshold on the AFB for our use case scenario we searched the literature for association between the AFB and the development of strokes. Specifically, Boriani et al.^32^ showed that an individual with an AFB greater than 4% will have increased prognostic of having a stroke. We thus decided to settle our decision threshold at 4%.

### Reporting summary

Further information on research design is available in the Nature Research Reporting Summary linked to this article.

## Supplementary information

Supplement

REPORTING SUMMARY

## Data Availability

The data that support the findings of this study included raw ECG and manual beat labels for atrial tachyarrhythmia as well as demographic information. Data may be made available for noncommercial academic use from the authors with permission from the respective hospitals they originated from. Please contact the corresponding author for such requests.

## Code availability

Source code for computing the heart rate variability and morphological features used in the XGB model are available on our open source platform at physiozoo.com. We are also working on integrating a version of ArNet2 on PhysioZoo.

## References

[1] Björck S, Palaszewski B, Friberg L, Bergfeldt L (2013). Atrial fibrillation, stroke risk, and warfarin therapy revisited: A population-based study. Stroke.

[2] Haim M (2015). Prospective national study of the prevalence, incidence, management and outcome of a large contemporary cohort of patients with incident non-valvular atrial fibrillation. J. Am. Hear. Assoc..

[3] Wolf PA, Abbott RD, Kannel WB (1991). Atrial fibrillation as an independent risk factor for stroke: The framingham study. Stroke.

[4] Shen AY-J (2010). Racial/ethnic differences in the prevalence of atrial fibrillation among older adults—a cross-sectional study. J. Natl. Med. Assoc..

[5] Chugh SS (2014). Worldwide epidemiology of atrial fibrillation: a Global Burden of Disease 2010 Study. Circulation.

[6] Pothineni NV, Vallurupalli S (2018). Gender and atrial fibrillation: differences and disparities. US Cardiol. Rev..

[7] Carrara M (2015). Heart rate dynamics distinguish among atrial fibrillation, normal sinus rhythm and sinus rhythm with frequent ectopy. Physiol. Meas..

[8] Moss TJ, Lake DE, Moorman JR (2014). Local dynamics of heart rate: detection and prognostic implications. Physiol. Meas..

[9] Chocron A (2021). Remote Atrial Fibrillation Burden Estimation Using Deep Recurrent Neural Network. IEEE Trans. Biomed. Eng..

[10] Wang J (2021). An intelligent computer-aided approach for atrial fibrillation and atrial flutter signals classification using modified bidirectional LSTM network. Inf. Sci. (Ny.)..

[11] Behar JA (2018). PhysioZoo: A Novel Open Access Platform for Heart Rate Variability Analysis of Mammalian Electrocardiographic Data. Front Physiol..

[12] Gendelman, S. et al. PhysioZoo ECG: Digital electrocardiography biomarkers to assess cardiac conduction. in *2021 Computing in Cardiology (CinC)* 48 1–4 (2021).

[13] Asgari S, Mehrnia A, Moussavi M (2015). Automatic detection of atrial fibrillation using stationary wavelet transform and support vector machine. Comput. Biol. Med..

[14] Petrenas A, Marozas V, Sörnmo L (2015). Low-complexity detection of atrial fibrillation in continuous long-term monitoring. Comput. Biol. Med..

[15] Granada J (2000). Incidence and predictors of atrial flutter in the general population. J. Am. Coll. Cardiol..

[16] Lake DE, Moorman JR (2011). Accurate estimation of entropy in very short physiological time series: the problem of atrial fibrillation detection in implanted ventricular devices. Am. J. Physiol. Circ. Physiol..

[17] Sarkar S, Ritscher D, Mehra R (2008). A detector for a chronic implantable atrial tachyarrhythmia monitor. IEEE Trans. Biomed. Eng..

[18] Mohanty S (2013). Results from a single-blind, randomized study comparing the impact of different ablation approaches on long-term procedure outcome in coexistent atrial fibrillation and flutter (APPROVAL). Circulation.

[19] Celikyurt U (2017). Incidence of new-onset atrial fibrillation after cavotricuspid isthmus ablation for atrial flutter. Ep Eur..

[20] Goldberger AL (2000). PhysioBank, PhysioToolkit, and PhysioNet: components of a new research resource for complex physiologic signals. Circulation.

[21] Wang, X. et al. Paroxysmal atrial fibrillation events detection from dynamic ECG recordings: The 4th China physiological signal challenge 2021. In *Proc. PhysioNet* 1–83 (2021).

[22] Kirchhof P (2016). 2016 ESC Guidelines for the management of atrial fibrillation developed in collaboration with EACTS. Kardiol. Pol..

[23] Link MS (2012). Evaluation and initial treatment of supraventricular tachycardia. N. Engl. J. Med..

[24] Link MS (2007). Introduction to the arrhythmias: a primer. EP Lab Dig..

[25] Kligfield P (2007). Recommendations for the standardization and interpretation of the electrocardiogram: part I: The electrocardiogram and its technology: a scientific statement from the American Heart Association Electrocardiography and Arrhythmias Committee, Council on Cli. Circulation.

[26] Pan J, Tompkins WJ (1985). A Real-Time QRS Detection Algorithm. IEEE Trans. Biomed. Eng. BME-.

[27] Clifford GD, Behar J, Li Q, Rezek I (2012). Signal quality indices and data fusion for determining clinical acceptability of electrocardiograms. Physiol. Meas..

[28] He, K., Zhang, X., Ren, S. & Sun, J. Deep residual learning for image recognition. In *Proceedings of the IEEE conference on computer vision and pattern recognition* 770–778 (2016).

[29] Kingma, D. P. & Ba, J. Adam: A method for stochastic optimization. In *Proc. 3rd International Conference on Learning Representations (ICLR)* (ICLR, 2015).

[30] Head, T., MechCoder, G. L., Shcherbatyi, I. & others. scikit-optimize/scikit-optimize: v0. 5.2. *Zenodo* (2018).

[31] Go AS (2018). Association of burden of atrial fibrillation with risk of ischemic stroke in adults with paroxysmal atrial fibrillation: the KP-RHYTHM study. JAMA Cardiol..

[32] Boriani G (2014). AF burden is important – fact or fiction?. Int. J. Clin. Pract..

