# Supplementary material for: Generalizable and robust deep learning algorithm for atrial fibrillation diagnosis across geography, ages and sexes
Source: NPJ Digit Med. 2023 Mar 17;6:44. doi: 10.1038/s41746-023-00791-1 (PMC10023682; doi:10.1038/s41746-023-00791-1)
Supplement: Supplementary file 1 — Supplement [file 41746_2023_791_MOESM1_ESM.pdf]

Supplementary notes

Supplementary Note 1: Benchmarking: 21 features from RR time interval in the context of AF detection have been identified in previous work by Chocron et al.[9], among which: CosEn[16], AFEv[17], PACEv[17] etc. The features were standardized by mean subtraction and division by the standard deviation extracted from the train set. Those features, extracted for individual 60-beat windows were integrated into state-of-the art classical machine learning method, XGB. XGB algorithm implemented in the *python XGBoost* library was used. ArNet developed in our previous work[9] consists of 1D-CNN network used as a feature extractor, further fed to a GRU pool. The number of estimator (n_e) as well as the maximal depth of the trees (m_d) for XGB and hyperparameter listed in Table S8 were optimized using 5-fold cross-validation on the train set.

Supplementary Figures

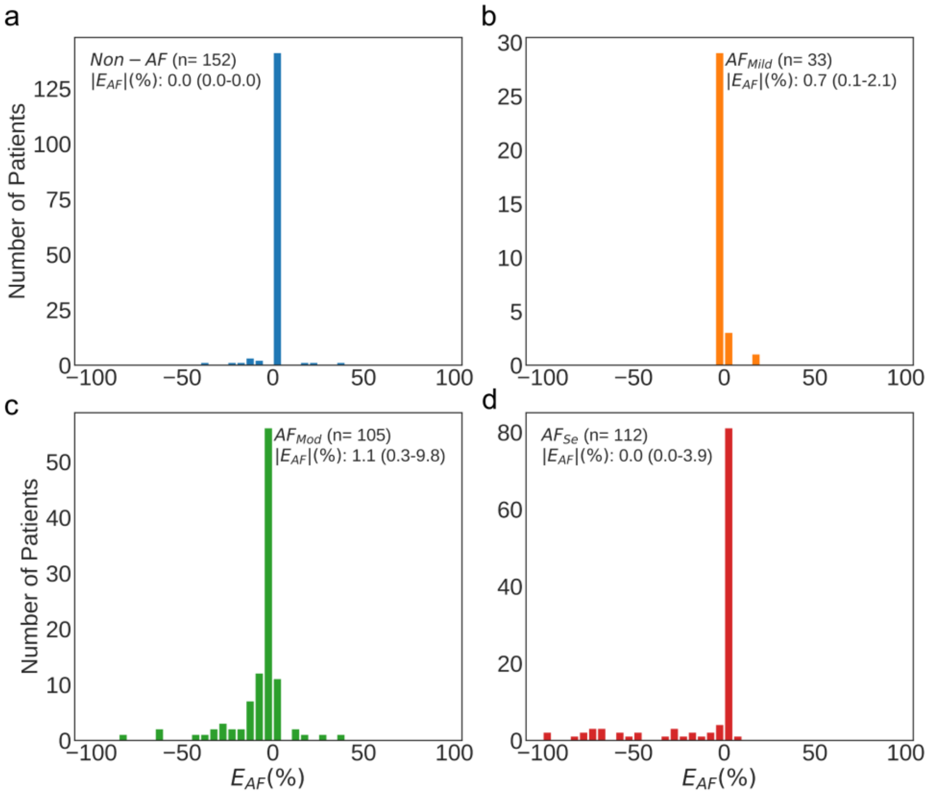

Supplementary Figure 1: $|E_{AF}(\%)|$ for the combined test set (n=402). The $|E_{AF}(\%)|$ is shown per different AF_l severity labels; Non-AF_l (panel a), AF_{mild} (Panel b), AF_{mod} (panel c) and AF_{sev} (panel d).

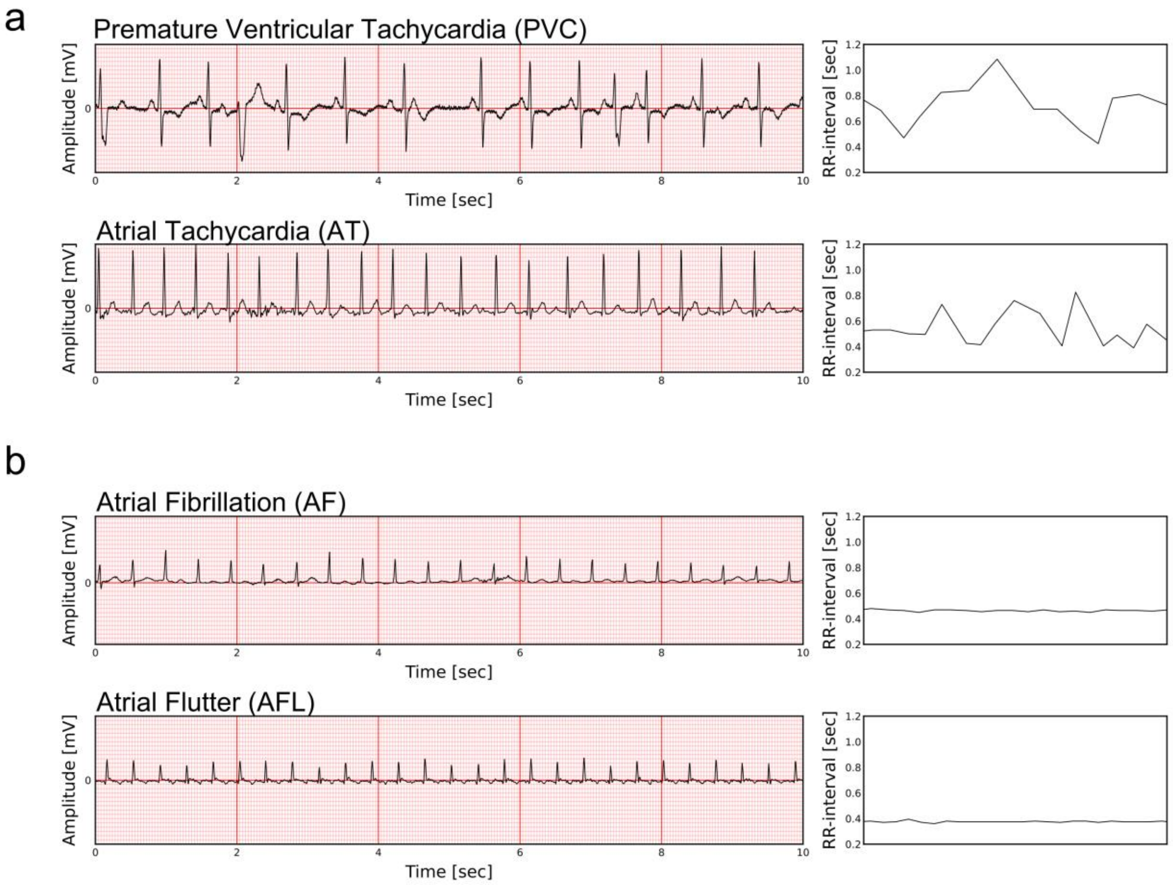

Supplementary Figure 2: Examples of misclassified windows. A representative short 10-sec of each window is presented followed by the window RR time series. FP examples (panel a) and FN (panel b).

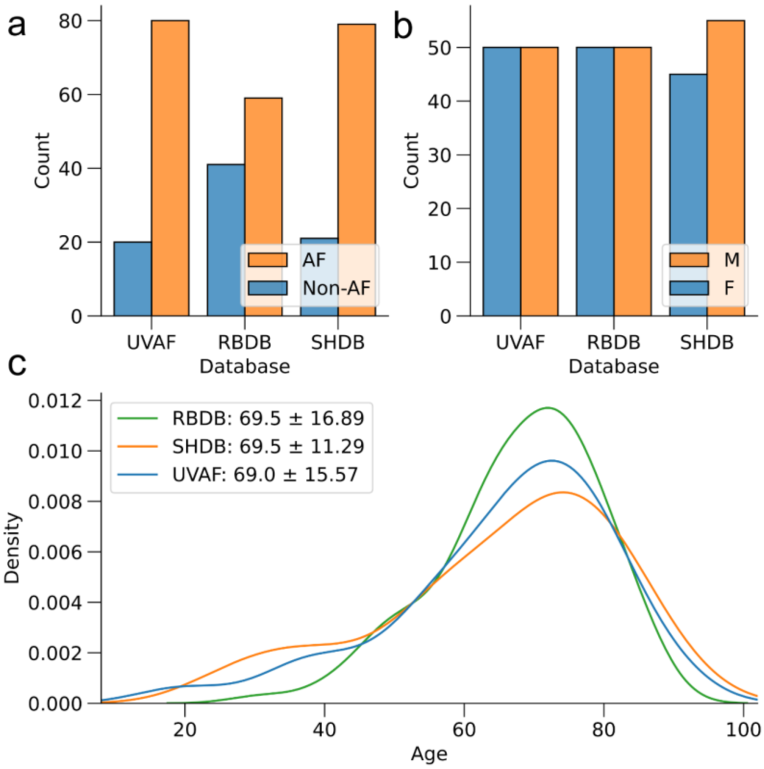

Supplementary Figure 3: Distribution for the re-annotated test sets. Diagnosis Label distribution (panel a), sex distribution (panel b) and age distribution (panel c) over the three geography groups test sets.

Supplementary Tables

		F_1	AUROC	Se	Sp	PPV	$\lvert E_{AF}(\%)\rvert$ μ (Q1 − Q3)
UVAF-train	AFEv	0.79	NA	0.79	0.99	0.79	5.90 (1.47-23.43)
	XGB	0.86	0.99	0.85	0.99	0.88	2.43 (0.52-11.29)
	ArNet	0.90	0.97	0.87	1.00	0.94	0.10 (0.0-6.27)
	ArNet2	0.92	1.00	0.93	0.99	0.91	0.15 (0.0-4.02)
UVAF-test	AFEv	0.90	NA	0.86	0.95	0.94	3.21 (1.28-10.72)
	XGB	0.92	0.97	0.88	0.96	0.96	1.42 (0.30-5.31)
	ArNet	0.93	0.98	0.89	0.97	0.97	0.08 (0.0-3.29)
	ArNet2	0.95	0.99	0.93	0.96	0.96	0.10 (0.0-2.41)
SHDB-test	AFEv	0.85	NA	0.79	0.97	0.92	0.91 (0.31-5.77)
	XGB	0.88	0.97	0.82	0.98	0.94	0.44 (0.13-4.86)
	ArNet	0.87	0.97	0.81	0.99	0.95	0.81 (0.23-3.73)
	ArNet2	0.92	0.99	0.89	0.98	0.94	0.59 (0.10-3.23)
RBDB-test	AFEv	0.80	NA	0.68	0.97	0.96	5.13 (1.05-28.79)
	XGB	0.81	0.95	0.70	0.98	0.97	1.59 (0.27-32.61)
	ArNet	0.85	0.98	0.74	0.99	0.99	0.36 (0.11-14.94)
	ArNet2	0.90	0.99	0.83	0.98	0.97	0.21 (0.09-7.59)
CPSC-test	AFEv	0.92	NA	0.91	0.96	0.93	6.76 (1.04-19.07)
	XGB	0.94	0.97	0.92	0.98	0.96	5.06 (0.62-19.03)
	ArNet	0.95	0.97	0.91	0.99	0.99	0.0 (0.0-12.78)
	ArNet2	0.95	0.99	0.95	0.97	0.94	0.0 (0.0-6.02)
Combined test set	AFEv	0.86	NA	0.79	0.97	0.94	2.73 (0.72-13.93)
	XGB	0.88	0.97	0.81	0.98	0.96	1.33 (0.25-9.09)
	ArNet	0.89	0.98	0.82	0.98	0.97	0.39 (0.02-6.11)
	ArNet2	0.92	0.99	0.89	0.97	0.96	0.32 (0.01-3.62)

Supplementary Table 1: Performance statistics for the UVAF-train, UVAF-test and external test sets, and the overall combined test set. For each performance measure the highest entry is underlined for per all geography group.

	True AFB (μ)		$\lvert E_{AF}(\%) \rvert$				
			Min	Q1	Median	Q3	Max
UVAF-train	0.0	AFEv	0.05	1.47	5.9	23.43	99.85
		XGB	0.0	0.52	2.43	11.29	95.68
		ArNet	0.0	0.0	0.1	6.27	100.0
		ArNet2	0.0	0.0	0.15	4.02	80.56
UVAF-test	24.49	AFEv	0.0	1.28	3.21	10.72	86.2
		XGB	0.0	0.30	1.42	5.31	81.73
		ArNet	0.0	0.0	0.08	3.29	83.56
		ArNet2	0.0	0.0	0.10	2.41	90.04
SHDB-test	6.73	AFEv	0.0	0.31	0.91	5.77	73.97
		XGB	0.0	0.13	0.44	4.86	72.67
		ArNet	0.03	0.23	0.81	3.73	70.78
		ArNet2	0.0	0.10	0.59	3.23	43.54
RBDB-test	9.67	AFEv	0.03	1.05	5.13	28.79	98.71
		XGB	0.0	0.27	1.59	32.61	97.00
		ArNet	0.02	0.11	0.36	14.94	97.79
		ArNet2	0.0	0.09	0.21	7.59	99.20
CPSC-test	0.0	AFEv	0.0	1.04	6.76	19.07	92.23
		XGB	0.0	0.62	5.06	19.03	53.76
		ArNet	0.0	0.0	0.0	12.78	67.67
		ArNet2	0.0	0.0	0.0	6.02	48.71
Combined test set	8.92	AFEv	0.0	0.72	2.73	13.93	98.71
		XGB	0.0	0.25	1.33	9.09	97.00
		ArNet	0.0	0.02	0.39	6.11	97.79
		ArNet2	0.0	0.01	0.32	3.62	99.20

Supplementary Table 2: $\lvert E_{AF}(\%) \rvert$ statistics across all geography groups, i.e for the UVAF train and test sets, and the generalization performance over all external test sets. Median (μ) and the interquartile range (Q1-Q3) are reported for the true AFB per test set.

	True AFB (μ)		$\lvert E_{AF}(\%) \rvert$				
			Min	Q1	Median	Q3	Max
Female	11.0	AFEv	0.0	0.95	3.19	13.47	93.68
		XGB	0.0	0.27	1.14	9.10	80.99
		ArNet	0.0	0.00	0.26	2.67	96.18
		ArNet2	0.0	0.00	0.14	2.46	99.20
Male	8.35	AFEv	0.0	0.58	2.2	15.77	98.71
		XGB	0.0	0.21	1.46	9.10	97.0
		ArNet	0.0	0.07	0.80	6.96	97.79
		ArNet2	0.0	0.06	0.71	4.79	93.36

Supplementary Table 3: $\lvert E_{AF}(\%) \rvert$ statistics for combined test set stratified by sex. Median (μ) and the interquartile range (Q1-Q3) are reported for the true AFB per test set.

		F_1	AUROC	Se	Sp	PPV	$\lvert E_{AF}(\%)\rvert$ μ (Q1 – Q3)
Female	AFEv	0.9	NA	0.84	0.98	0.97	3.19 (0.95-13.47)
	XGB	0.91	0.98	0.86	0.99	0.98	1.14 (0.27-9.10)
	ArNet	0.92	0.99	0.87	0.99	0.98	0.26 (0.0-2.67)
	ArNet2	0.94	0.99	0.92	0.98	0.97	0.14 (0.0-2.46)
Male	AFEv	0.82	NA	0.74	0.95	0.92	2.2 (0.58-15.77)
	XGB	0.84	0.95	0.76	0.97	0.94	1.46 (0.21-9.10)
	ArNet	0.86	0.86	0.97	0.78	0.98	0.80 (0.07-6.96)
	ArNet2	0.90	0.98	0.86	0.97	0.95	0.71 (0.06-4.79)

Supplementary Table 4: Performance statistics for the combined test set divided by sex. For each performance measure the highest entry is underlined for per sex.

	True AFB (μ)		$\lvert E_{AF}(\%)\rvert$				
			Min	Q1	Median	Q3	Max
≤ 60	0.0	AFEv	0.0	0.46	1.14	7.51	96.86
		XGB	0.0	0.19	0.67	4.27	92.52
		ArNet	0.0	0.0	0.26	3.62	84.31
		ArNet2	0.0	0.0	0.14	2.81	41.92
61 to 75	16.01	AFEv	0.0	0.55	1.92	13.68	98.71
		XGB	0.0	0.16	1.13	10.63	82.84
		ArNet	0.0	0.09	0.89	9.89	96.18
		ArNet2	0.0	0.05	0.47	5.15	99.20
> 75	46.71	AFEv	0.0	1.43	4.24	15.89	98.45
		XGB	0.0	0.61	2.03	9.09	97.0
		ArNet	0.0	0.0	0.24	3.81	97.79
		ArNet2	0.0	0.0	0.22	2.66	93.36

Supplementary Table 5: $\lvert E_{AF}(\%)\rvert$ statistics for combined test set grouped into three different age groups. Median (μ) and the interquartile range (Q1-Q3) are reported for the true AFB per test set.

		F_1	AUROC	Se	Sp	PPV	$\lvert E_{AF}(\%)\rvert$ μ (Q1 – Q3)
≤ 60	AFEv	0.83	NA	0.77	0.98	0.91	1.14 (0.46-7.51)
	XGB	0.87	0.98	0.80	0.99	0.94	0.67 (0.19-4.27)
	ArNet	0.90	0.99	0.83	1.00	0.99	0.26 (0.0-3.62)
	ArNet2	0.95	1.00	0.93	0.99	0.97	0.14 (0.0-2.81)
61 to 75	AFEv	0.84	NA	0.75	0.98	0.96	1.92 (0.55-13.68)
	XGB	0.86	0.96	0.77	0.98	0.97	1.13 (0.16-10.63)
	ArNet	0.86	0.97	0.77	0.98	0.97	0.89 (0.09-9.89)
	ArNet2	0.90	0.98	0.86	0.97	0.96	0.47 (0.05-5.15)
> 75	AFEv	0.88	NA	0.83	0.93	0.94	4.24 (1.43-15.89)
	XGB	0.90	0.95	0.85	0.94	0.96	2.03 (0.61-9.09)
	ArNet	0.92	0.98	0.88	0.96	0.97	0.24 (0.0-3.81)
	ArNet2	0.94	0.98	0.91	0.94	0.96	0.22 (0.0-2.66)

Supplementary Table 6: Performance statistics for the combined test set grouped into three different age groups. For each performance measure the highest entry is underlined for per age group.

		Non-AF_l	AF_{mild}	AF_{mod}	AF_{sev}
geography	UVAF-test	0.0, 0.0-0.03 (27)	0.85, 0.28-2.18 (8)	1.77, 0.18-5.8 (22)	0.0, 0.0-1.48 (43)
	SHDB-test	0.0, 0.0-0.0 (21)	0.61, 0.28-2.51 (23)	0.47, 0.08-2.43 (49)	8.42, 1.95-17.28 (7)
	RBDB-test	0.0, 0.0-0.0 (43)	7.55, 4.71-10.4 (2)	0.19, 0.07-0.63 (23)	0.18, 0.09-11.56 (32)
	CPSC-test	0.0, 0.0-0.0 (51)	6.45, 4.43-16.69 (24)	NA (0)	0.0, 0.0-0.0 (27)
	Combined test set	0.0, 0.0-0.0 (142)	2.11, 0.4-6.2 (57)	0.43, 0.1-2.55 (94)	0.04, 0.0-3.74 (109)
Sex	Female	0.0, 0.0-0.0 (75)	1.31, 0.34-6.08 (16)	0.17, 0.06-1.54 (41)	0.02, 0.0-3.27 (54)
	Male	0.0, .0-0.0 (67)	3.23, 0.57-6.33 (40)	0.86, 0.27-2.66 (53)	0.04, 0.0-3.22 (55)
Ages	≤ 60	0.0, 0.0-0.0 (69)	3.62, 0.08-6.7 (13)	0.23, 0.05-1.85 (16)	0. 0, 0.0-1.31 (18)
	61 to 75	0.0, 0.0-0.0 (46)	1.90, 0.46-8.2 (29)	0.42, 0.08-2.48 (42)	0.0, 0. 0-11.17 (39)
	> 75	0.0, 0.0-0.0 (27)	2.87, 0.72-5.29 (15)	0.79, 0.16-2.58 (36)	0.04, 0.0-2.6 (18)

Supplementary Table 7: $|E_{AF}(\%)|$ statistics for ArNet2 for internal test set, external test sets and age and ages groups per different AF_l severity labels; Non-AF_l, AF_{mild}, AF_{mod} and AF_{sev}. Median (μ) and the interquartile range (Q1-Q3) are reported and separated by a comma. The number of recordings in each group is reported in parenthesis.

Hyperparameter	Type	Range	Prior
w_s	Categorical	[60, 70, 80, 90, 100, 110, 120]	N.A.
n_b	Integer	[3, 7]	Uniform
n_f	Integer	$[2^5, 2^7]$	Log uniform
f_l	Integer	[3, 10]	Uniform
d_{r1}	Real/Continuous	[0, 0.5]	Uniform
n_d	Integer	$[2^6, 2^9]$	Log uniform
d_{r2}	Real/Continuous	[0, 0.8]	Uniform
α	Real/Continuous	$[10^{-5}, 10^{-2}]$	Log uniform
h	Integer	$[3, 20]$	Uniform

Supplementary Table 8: Hyperparameter search space for Bayesian optimization. for Uniform and Log uniform distribution the range is given with (lower, upper) bounds.
