# Supplementary material for: Generalizable and robust deep learning algorithm for atrial fibrillation diagnosis across geography, ages and sexes
Source: NPJ Digit Med. 2023 Mar 17;6:44. doi: 10.1038/s41746-023-00791-1 (PMC10023682; doi:10.1038/s41746-023-00791-1)
Supplement: Supplementary file 2 — REPORTING SUMMARY [file 41746_2023_791_MOESM2_ESM.pdf]

nature research

Corresponding author(s): Joachim A. Behar

Reporting Summary

Nature Research wishes to improve the reproducibility of the work that we publish. This form provides structure for consistency and transparency in reporting. For further information on Nature Research policies, see Authors & Referees and the Editorial Policy Checklist.

Statistical parameters

When statistical analyses are reported, confirm that the following items are present in the relevant location (e.g. figure legend, table legend, main text, or Methods section).

n/a	Confirmed	
☐	☒	The exact sample size (*n*) for each experimental group/condition, given as a discrete number and unit of measurement
☐	☒	An indication of whether measurements were taken from distinct samples or whether the same sample was measured repeatedly
☐	☒	The statistical test(s) used AND whether they are one- or two-sided<br>*Only common tests should be described solely by name; describe more complex techniques in the Methods section.*
☒	☐	A description of all covariates tested
☒	☐	A description of any assumptions or corrections, such as tests of normality and adjustment for multiple comparisons
☐	☒	A full description of the statistics including central tendency (e.g. means) or other basic estimates (e.g. regression coefficient) AND variation (e.g. standard deviation) or associated estimates of uncertainty (e.g. confidence intervals)
☐	☒	For null hypothesis testing, the test statistic (e.g. *F*, *t*, *r*) with confidence intervals, effect sizes, degrees of freedom and *P* value noted<br>*Give P values as exact values whenever suitable.*
☒	☐	For Bayesian analysis, information on the choice of priors and Markov chain Monte Carlo settings
☒	☐	For hierarchical and complex designs, identification of the appropriate level for tests and full reporting of outcomes
☒	☐	Estimates of effect sizes (e.g. Cohen's *d*, Pearson's *r*), indicating how they were calculated
☒	☐	Clearly defined error bars<br>*State explicitly what error bars represent (e.g. SD, SE, CI)*

Our web collection on statistics for biologists may be useful.

Software and code

Policy information about availability of computer code

Data collection	Philips Holter software, Fukuda Holter monitor and PathFinder Holter monitor were used to collect the ecg recordings.
Data analysis	Our open source platform at physiozoo.com was used to extract heart rate variability and morphological features. Python libraries were used for ecg filtering and analysis such as scipy, wfdb, MNE-Python and pandas.

For manuscripts utilizing custom algorithms or software that are central to the research but not yet described in published literature, software must be made available to editors/reviewers upon request. We strongly encourage code deposition in a community repository (e.g. GitHub). See the Nature Research guidelines for submitting code & software for further information.

Data

Policy information about availability of data

All manuscripts must include a data availability statement. This statement should provide the following information, where applicable:

- Accession codes, unique identifiers, or web links for publicly available datasets
- A list of figures that have associated raw data
- A description of any restrictions on data availability

The data that support the findings of this study included raw ECG and manual beat labels for atrial tachyarrhythmia as well as demographic information. Data may be made available for noncommercial academic use from the authors with permission from the respective hospitals they originated from. Please contact the

March 2018

corresponding author for such requests.
Source code for computing the heart rate variability and morphological features used in the XGB model are available on our open source platform at physiozoo.com

Field-specific reporting

Please select the best fit for your research. If you are not sure, read the appropriate sections before making your selection.

☒ Life sciences ☐ Behavioural & social sciences

For a reference copy of the document with all sections, see nature.com/authors/policies/ReportingSummary-flat.pdf

Life sciences

Study design

All studies must disclose on these points even when the disclosure is negative.

Sample size	4,298 Holter recordings
Data exclusions	Holter recordings of patients under the age of 18 years were excluded since this research focused on AF diagnosis in adults. Furthermore, corrupted recordings were also excluded.<br>Regarding the UVAF train set, some reference beat labels were missing. To account for that, windows with over 10 seconds of missing beat labels were excluded. Patients presenting over 25% of 60-beat windows with missing beat labels based on this criterion were discarded. Among the remaining recordings, windows with bSQI lower than 0.8 were considered of low quality and were excluded. Recordings with a rate of exclusion, i.e. the number of excluded windows over the total number of windows exceed 75% were considered as corrupted by noise and were discarded.<br>No recordings were excluded from the test sets.<br>Sex was not reported for a single patient in CPSC-test and thus we excluded this recording from the sex group.
Replication	A total of 100 recordings were selected from the original databases while stratifying by age, sex and diagnosis for AFl. Specifically, 80 recordings from patients that had AF based on the cardiology report were selected for each test set. For the RBDB-test and SHDB-test, the AF diagnoses were obtained from the medical report prepared following the patient's examination. As no patient reports were available for UVAF, the diagnosis was inferred from the AF events annotated in the recordings. For the CPSC-test the complete available open dataset was used as is.
Randomization	No randomization was performed during allocation to experimental (i.e geography, sex and age) groups.
Blinding	*Describe whether the investigators were blinded to group allocation during data collection and/or analysis. If blinding was not possible, describe why OR explain why blinding was not relevant to your study.*

Materials & experimental systems

Policy information about availability of materials

n/a	Involved in the study
☒	☐ Unique materials
☒	☐ Antibodies
☒	☐ Eukaryotic cell lines
☒	☐ Research animals
☐	☒ Human research participants

Human research participants

Policy information about studies involving human research participants

Population characteristics	Patients referred for an Holter examination at cardiology clinics. Retrospective data analysis

Method-specific reporting

n/a	Involved in the study
☒	☐ ChIP-seq
☒	☐ Flow cytometry
☒	☐ Magnetic resonance imaging
